# Short-term outcomes of near-infrared imaging using indocyanine green in laparoscopic lateral pelvic lymph node dissection for middle-lower rectal cancer: A propensity score-matched cohort analysis

**DOI:** 10.3389/fmed.2022.1039928

**Published:** 2022-11-10

**Authors:** Jin-Yu Dai, Zhi-Jun Han, Jing-Dong Wang, Bao-Shuang Liu, Jian-Yu Liu, Yan-Cheng Wang

**Affiliations:** Department of Enterochirurgia, Fengrun District People's Hospital, Tangshan, China

**Keywords:** rectal cancer, lateral pelvic lymph node dissection (LPLD), 3D laparoscopic technology, indocyanine green, post-operative complications

## Abstract

Laparoscopic lateral pelvic lymph node dissection (LPND) is limited by complex neurovascular bundles in the narrow pelvic sidewall and various post-operative complications. Indocyanine green (ICG) has been applied to increase the number of harvested lymph nodes and reduce the injury of irrelevant vessels in patients with rectal cancer. However, few studies on the recurrence rate of ICG fluorescence imaging-guided laparoscopic LPND were reported. This retrospective study enrolled 50 middle- low rectal cancer patients who were treated by LPND. After propensity score matching, 20 patients were matched in each of the indocyanine green (ICG) guided imaging group (ICG group) and non-ICG guided imaging group (non-ICG group). The average follow-up time was 13.5 months (12–15 months). Our results showed that the total number of harvested lymph nodes in the ICG group was significantly higher than that in the non-ICG group (*P* < 0.05), and intraoperative blood loss and post-operative hospital stay times in the ICG group were less than those in the non-ICG group (*P* < 0.05). After 12 months of follow-up, no residual lymph node and local tumor recurrence were found for patients in the ICG group. Four patients in the non-ICG group detected residual lymph nodes at the 3-month visit. Our findings highlighted the importance of ICG fluorescence-guided imaging in LPND because it has unique advantages in improving the number of lymph node dissections, surgical accuracy, and decreasing the residual lymph nodes and local tumor recurrence. In addition, ICG fluorescence guidance technology can effectively shorten the operation time, and it is simple to operate, which is worth popularizing.

## Introduction

Lateral pelvic lymph node metastasis (LPNM) was related to local recurrence after radical resection due to a high incidence of LPNM in patients with middle and low rectal cancer (1, 2). As a standard treatment, pre-operative chemoradiotherapy (CRT) plus total mesorectal excision (TME) has been recommended for patients with advanced rectal cancer with or without LPNM in western countries (3, 4). However, lateral local recurrence is still a significant problem after pre-operative chemoradiotherapy (CRT) plus total mesorectal excision (TME) in patients with enlarged lateral pelvic lymph nodes (LPLNs) (5). Ogura et al. (5) discovered that the application of LPLN dissection (LPND) results in a significantly lower lateral local recurrence rate in patients with low cT3/4 rectal cancer. The latest Japanese Society for Cancer of the Colon and Rectum (JSCCR) guidelines recommends that LPND is an effective treatment strategy for patients with LPNM (2, 6, 7). Thus far, the safety and feasibility of LPND for rectal cancer with clinical evidence of LPNM has been widely confirmed by other Asian countries, such as China, and South Korea (8–11).

Laparoscopic LPND is limited by various complications from the viewpoint of complex neurovascular bundles of the lateral region in the pelvis (12). Clinically, complications related to Laparoscopic LPND without efficient guidance were significantly higher than simple TME, such as autonomic nerve dysfunction, hypogastric nerves, and ureter damage (13). In addition, the tissue edema and adhesion caused by pre-operative CRT increase the difficulties of LPND in the narrow pelvic sidewall (14). As a result, laparoscopic LPND is technically challenging (8). Previous studies have indicated that laparoscopic LPND showed more safety in intraoperative blood loss and post-operative complications than open surgery, (15, 16) however, significant curative effect differences of LPND in patients with clinical evidence of LPNM remain cannot be ignored due to the lack of a unified standard of LPND (13, 17, 18). Therefore, the application of LPND is still limited (19, 20).

As an inexpensive and safe non-specific fluorescent probe, indocyanine green (ICG) has been applied to improve sentinel lymph node detection, (21–23) to reduce post-operative anastomotic leakage (24–27). In LPND protocol for patients with rectal cancer, ICG bonds rapidly to serum proteins and migrates to lymphatic vessels when injected topically into the intestinal submucosa; the lymphatic flow could be visualized by near-infrared imaging using ICG fluorescence. On this basis, ICG-guided LPND has been reported to reduce the injury of irrelevant vessels and increase the number of harvested lymph nodes in the pelvis (28–31). There are few studies on the recurrence rate of ICG fluorescence imaging-guided laparoscopic LPND in patients with middle and low rectal cancer. The purpose of this study is to explore the safety and effectiveness of ICG fluorescence imaging-guided laparoscopic LPND in patients with middle and low rectal cancer by comparing the surgical results and tumor recurrence within 1 year after laparoscopic LPND with and without ICG fluorescence imaging.

## Patients and methods

In this longitudinal study, we retrospectively reviewed the consecutive records of all patients with middle-lower rectal cancer who visited the Fengrun District People's Hospital of Tangshan (Tangshan, Hebei, province, China) between February 2018 and February 2019. This study was approved and waived the requirement for consent due to the retrospective nature by the ethics committee of Fengrun District People's Hospital of Tangshan. The study protocol adhered to the tenets of the Declaration of Helsinki.

### Study subjects

All patients underwent pre-operative routine physical examinations; blood routine examination; hepatorenal function test; serum carcinoembryonic antigen (CEA); chest and abdominal X-rays. Tumor evaluation included a total colonoscopy, endorectal ultrasonography, whole-body computed tomography (CT), digital rectal examination, and Pelvic magnetic resonance imaging (MRI). Then, pre-operative staging diagnoses were made for all patients, according to American Joint Commission on Cancer staging system (AJCC, 8th Edition). For patients with large tumor diameters or with many LPNMs, experienced surgeons should decide whether pre-operative CRT was performed. Laparoscopic TME plus LPND (with or without ICG guided imaging) should be conducted 6–8 weeks after CRT.

Inclusion criteria were as follows: (1) patients aged over 20 years, diagnosed with middle and low rectal cancer by pre-operative routine physical examinations and pathological examination; (2) clinical evidence of LPNM based on pre-operative MRI; (3) the distance between a tumor and anal verge was within 80 mm; (4) with follow-up time longer than 12 months.

Exclusion criteria were as follows: patients with (1) abdominal adhesion, or (2) recurrent rectal cancer, or (3) distant metastasis, or (4) resection at two or more primary sites are required, or (5) emergency surgery is required due to acute intestinal perforation and acute intestinal obstruction, or (6) Iodine allergy.

Lateral pelvic lymph node metastasis was defined as follows (8): (1) with 5mm or longer short axial diameter; (2) non-homogeneous or intense enhancement, and (3) blurred edge. An experienced radiologist and an oncologist confirmed LPNM. Pathological specimens were also examined by two pathologists specializing in colorectal cancer.

### Laparoscopic LPND procedure

According to the radical principle of rectal cancer, five ports are generally used in laparoscopic surgery. After total mobilization of the rectum and distal rectal transection, LPND was performed in these regions: internal iliac lymph node, obturator lymph node, external iliac lymph node, and common iliac lymph node (6). First, the ureter, hypogastric, and pelvic nerves were isolated from the pelvic sidewall to avoid injuring these structures. Then, LPND was performed in order along the external iliac vessels, the common iliac vessels and the obturator fossa, and the internal iliac vessels. Special care was required to preserve the autonomic and obturator nerves and the superior vesical artery. If metastatic lymph nodes invaded these arteries, these vessels and lymph nodes were cleared to ensure adequate safety boundaries.

### ICG-guided imaging

Laparoscopic lateral pelvic lymph node dissection was guided by the laparoscopic near-infrared imaging system (Karl Storz Endoscope spies TM GmbH & Co. KG, Tuttlingen, Germany). After anesthesia, a total of 1.0 ml of ICG (2.5 mg/ml) was injected into the rectal submucosa on the anal side of the tumor through a fine needle (4.5 gauge). During LPND, the laparoscopic light source was switched to green light *via* a footswitch. Then lymph nodes were distinguished and dissected thoroughly from the non-lymphatic soft tissue by ICG fluorescence-guided imaging to prevent unnecessary damage and lymph node omission.

### Non-ICG guided imaging

Patients without ICG-guided imaging received the same surgical procedure as those in the ICG-guided imaging group but did not use ICG -guided imaging for LPND.

HZJ, skilled in laparoscopic surgery, performed all laparoscopic TME plus LPND (with or without ICG) procedures. After dissection, tumor and lymph node specimens were verified by pathological examination.

### Main outcomes

Pre-operative demographic characteristics and baseline data were compared between groups, such as age, sex, BMI, ASA, pre-operative CEA level, tumor height from the anal verge, tumor diameter, and TNM clinical stage. Operation-related outcomes included harvested lateral pelvic lymph nodes, operation time, intraoperative blood loss, intraoperative blood transfusion rate, conversion to open surgery rate, post-operative hospital stay times, and all post-operative complications within 30 days after the operation [Clavien–Dindo classification (CD) grade ≥II].

### Follow-up

All patients were followed up every 3 months within 3 years after hospital discharge. Serum tumor marker analysis and chest and abdominal CT examination were performed every 6 months. Local recurrence and residual lymph nodes in the Lateral pelvic were recorded until April 30, 2022.

### Data analysis

Propensity score matching was used 1:1 to match patients in the ICG group with those in the non-ICG group (caliper = 0.2). Age, sex, and BMI were matching factors. Differences in continuous variables were compared using a *t*-test, and data were shown as the mean ± standard deviation. Categorical variables were compared using the Chi-squared test or Fisher exact test and were shown as frequencies and percentages. Differences were considered statistically significant when *P* < 0.05.

## Results

Overall, 50 middle- low rectal cancer patients with clinical evidence of LPNM who underwent TME plus LPND were enrolled from February 2018 to February 2019 at Fengrun District People's Hospital of Tangshan. The average follow-up time was 13.5 months (12–15 months). Among them, 26 patients were treated with ICG-guided imaging. After propensity score matching, 40 patients were matched in each of the ICG group (*n* = 20) and non-ICG group (*n* = 20).

### Comparison of baseline characteristics

There was no significant difference in age, sex, BMI, ASA, concomitant pre-operative disease (diabetes, hypertension, atherosclerosis), pre-operative CEA level, tumor height from the anal verge, tumor diameter, and TNM clinical stage between the two groups (Tables 1, 2).

**Table 1:** Comparison of baseline characteristics.

**Variables**	**ICG (*n* = 20)**	**non-ICG (*n* = 20)**	***t/X^2^***	***P*-value**
Age, (years)	63 ± 9.0	62 ± 9.4	0.343	0.733
**Gender**, ***n*** **(%)**			0.102	0.749
Male	12 (60%)	11 (55%)		
Female	8 (40%)	9 (45%)		
BMI, (kg/m^2)^	22.3 ± 4	21.5 ± 5	0.559	0.580
Concomitant disease, *n* (%)	10 (50%)	9 (45%)	0.100	0.752
**ASA class**, ***n*** **(%)**			0.559	0.756
I	4 (20%)	6 (30%)		
II	11 (55%)	10 (50%)		
III	5 (25%)	4 (20%)		
Pre-operative chemoradiotherapy, *n* (%)	3 (15%)	4 (20%)	0.173	0.677

ICG, indocyanine green. Values are shown as Mean ± SD or %.

**Table 2:** Comparison of tumor characteristics.

**Variables**	**ICG (*n* = 20)**	**non-ICG (*n* = 20)**	***t/X^2^***	***P*-value**
**TNM**			0.125	0.723
II, *n* (%)	5 (25%)	6 (30%)		
III, *n* (%)	15 (75%)	14 (70%)		
**pT stage**, ***n*** **(%)**			0.594	0.898
I	4 (20%)	5 (25%)		
II	5 (25%)	6 (30%)		
III	9 (45%)	8 (40%)		
IV	2 (10%)	1 (5%)		
**pN stage**, ***n*** **(%)**			0.150	0.928
0	5 (25%)	6 (30%)		
I	6 (30%)	6 (30%)		
II	9 (45%)	8 (40%)		
CEA	4.4 ± 1.9	4.1 ± 1.7	0.526	0.602
Tumor size, (cm, mean ± SD)	4.1 ± 1.4	3.6 ± 1.1	1.256	0.217
**Distance from anal verge**			0.125	0.723
Low (<5 cm), *n* (%)	6 (30%)	5 (25%)		
Mid (5–8 cm), *n* (%)	14 (7%)	15 (75%)		

ICG, indocyanine green. Values are shown as Mean ± SD or %.

### Comparison of operation-related outcomes

Table 3 summarizes the operation-related outcomes. Regarding the type of operation, sphincter preservation, diverting stoma, autonomic nerve preservation, combined resection of adjacent organs, no significant differences were observed between the groups. Compared with the non-ICG group, the total number of harvested LPLDs was higher in the ICG group (19.2 ± 6.6 vs. 15.0 ± 4.6, *P* = 0.024); the operation time of the ICG group was longer than that of the non-ICG group (386 ± 45 vs. 332 ± 48 min, *P* = 0.001), and the intraoperative blood loss (22 ± 9 vs. 89 ± 14 ml, *P* < 0.001) and the post-operative hospital stay was lower (6 ± 2.2 vs. 8 ± 3.4 days, *P* = 0.033) in ICG group; no significant difference was found in the rate of intraoperative blood transfusion between the two groups (*P* = 0.548) (Table 3).

**Table 3:** Comparison of operation outcomes.

**Variables**	**ICG (*n* = 20)**	**non-ICG (*n* = 20)**	***t/X^2^***	***P*-value**
**Type of operation**, ***n*** **(%)**			0.229	0.633
Low anterior resection	17 (85%)	18 (90%)		
Abdominoperineal resection	3 (15%)	2 (10%)		
Sphincter preservation, *n* (%)	17 (85%)	16 (80%)	–	1.000*
Diverting stoma, *n* (%)	17 (85%)	18 (90%)	–	1.000*
Autonomic nerve preservation, *n* (%)	20 (100%)	20 (100%)	–	1.000*
Combined resection of adjacent organs, *n* (%)	1 (5%)	2 (10%)	–	1.000*
Number of harvested LPLNs	19.2 ± 6.6	15.0 ± 4.6	2.335	0.024
Unilateral dissection, *n* (%)	7 (35%)	6 (30%)	0.114	0.736
Bilateral dissection, *n* (%)	13 (65%)	14 (70%)	0.114	0.736
Conversion to laparotomy, *n* (%)	0 (0)	2 (10%)	–	0.147*
Operation time, (min)	386 ± 45	332 ± 48	3.670	0.001
Intraoperative blood loss, (ml)	22 ± 9	89 ± 14	18.003	<0.001
Intraoperative blood transfusion, *n* (%)	1 (5%)	2 (10%)	0.360	0.548
Post-operative hospital stay (days)	6 ± 2.2	8 ± 3.4	2.209	0.033
Total post-operative complication (CD grade ≥II), *n* (%)	8 (40%)	11 (55%)	0.902	0.342
Anastomotic leakage, *n* (%)	1 (5%)	2 (10%)	0.360	0.548
Infection, *n* (%)	3 (15%)	3 (15%)	0.000	1.000
Urinary retention, *n* (%)	0 (0)	2 (10%)	–	0.147*
Bowel obstruction, *n* (%)	2 (10%)	4 (20%)	0.784	0.376
Local recurrence, *n* (%)	0 (0)	2 (10%)	–	0.147*
Residual lymph nodes after LPND, *n* (%)	0 (0)	4 (20%)	–	0.035*

ICG, indocyanine green; LND, lymph node dissection; LPLNs, lateral pelvic lymph node; LPND, LPLN dissection. Values are shown as Mean ± SD or %. *Comparison was performed using Fisher exact test.

In total, there were 27 patients underwent bilateral dissection (13 patients in the ICG group and 14 patients in the non-ICG group, *P* = 0.736). No patient was converted to laparotomy in the ICG group, and two patients were converted to laparotomy in the non-ICG group due to intraoperative injury of the internal iliac vein and obturator artery, although the difference was not significant (*P* = 0.147). Intraoperative and post-operative pathology showed no positive circumference resection margin or distal resection margin in both groups.

Total post-operative complications (CD grade ≥II) did not differ to a statistically significant extent (the rate was 40 and 55% in the ICG group and non-ICG group, respectively, *P* = 0.342), although, more patients with various complications were found in the non-ICG group: two cases suffered from the anastomotic leakage, and were cured by washing and drainage; three cases with wound infection, two cases with urinary retention and four cases with bowel obstruction. no significant difference in grade ≥III complications.

### Comparison of follow-up outcomes

At the 3 months visit, four patients' CT images in the non-ICG group detected enlarged lymph nodes in the right internal iliac region, which suggested that they were residues in LPND; rectal cancer cells invasions were found based on the post-operative pathology in two patients who accepted further dissection of residual lymph nodes. Two patients declined further dissection of residual lymph nodes, and both two cases developed local tumor recurrence during 12 months visit. During follow-up, no residual lymph nodes and tumor recurrence were found in the ICG group.

## Discussion

We compared the differences in the number of harvested LPLNs, intraoperative blood loss, post-operative complications, and short-term local recrudescence between ICG and non-ICG groups in the current study. Our results showed that the total number of harvested LPLNs in the ICG group was significantly higher than that in the non-ICG group, and intraoperative blood loss and post-operative hospital stay in the ICG group were less than those in the non-ICG group. After 12 months of follow-up, no residual lymph node and local tumor recurrence were found for patients in the ICG group. Four patients in the non-ICG group detected residual lymph nodes at the 3-month visit, rectal cancer cell invasions were found in two patients who accepted further dissection of residual lymph nodes, and both two patients who declined further dissection of residual lymph nodes developed local tumor recurrence at 12 months visits.

The pelvic lateral wall is narrow, and Laparoscopic LPND operation is considered a challenging surgical technique (8). ICG fluorescence-guided imaging technology makes lymphatic flow visible and improves the clearance rate of suspicious lymph nodes. Consistent with previous studies, in the current results, ICG fluorescence-guided imaging helps to increase the number of harvested LPLNs than non-ICG fluorescence-guided Laparoscopic LPND operation. The intraoperative blood loss in the ICG group was less than that in the non-ICG group. In addition, although there is no significant difference between the two groups in the total post-operative complications with CD grade ≥II, the rate of anastomotic leakage, bowel obstruction, and urinary retention was higher in the non-ICG group, which may be related to the fact that the post-operative hospital stay in the non-ICG group is more extended than that in the ICG group. ICG fluorescence-guided imaging technology improves the tissue signal background ratio. It visualizes the lymphatic flow through local injection of ICG, which improves the recognition rate of lymph nodes and helps surgeons distinguish lymphatic vessels and lymph nodes from non-lymphatic soft tissues. It can decrease incorrect operations at the wrong level and reduce unnecessary damage to vessels and nerves during the process. This is the basic principle to avoid accidental bleeding and damage during LPND (2, 30).

Lateral pelvic lymph node metastasis is an important metastasis pathway of middle and low rectal cancer (1, 2). Sugihara et al. (32) found that the overall survival rate of patients with LPNM was significantly lower than that of patients with negative LPNM. The clinical study of JCOG0212 in Japan showed that the recurrence rate of patients with stage II/III rectal cancer after TME surgery was 13%, while the recurrence rate of patients with LPNM was 56.8% (19, 33). Ogura et al. (5) reported that suspicious lymph nodes were found in 58% of patients with low rectal cancer, which MRI confirmed, and the 5-year pelvic local recurrence rate was 5.5%. Studies suggest that local tumor recurrence after TME plus LPND was related to incomplete lymph node dissection, and effective LPND can improve the prognosis of rectal cancer patients (34). In the current research, we found that in the non-ICG group, CT Reexamination 3 months after operation showed that four patients had pelvic lymph node residues. After a 1-year follow-up, both two patients without further dissection of residual lymph nodes developed local tumor recurrence. Therefore, ICG fluorescence-guided laparoscopic LPND can provide effective guidance for detecting concealed LPLNs and reduce the risk of local recurrence within 1 year after the operation. We recommend further dissection of residual lymph nodes for patients with residual LPNM shown in CT or MRI, which can bring better short-term oncological outcomes for middle and low rectal cancer (2, 30, 31).

Identification of LPNM was evaluated traditionally by abdominoperineal CT or pelvic MRI. However, these methods are limited in distinguishing specific lymph nodes in actual surgery. In the current study, no residual lymph node was found in the ICG group. These results confirmed that ICG could effectively improve the development of lymph nodes, improve the rate of LPND, and reduce the possibility of metastasis. However, it should be noted that ICG helps enhance the development of lymph flow, but it is weak in distinguishing metastatic lymph nodes from normal lymph nodes. Usually, due to a large number of cancer cells in the metastatic lymph nodes, the density is uneven, which may also cause a decrease in fluorescence development. This is the limitation of ICG.

Limitations are found in this study. First, it was retrospective research, selection bias cannot be ruled out. However, we used propensity score matching to minimize selection bias. Second, the sample size is relatively small, resulting in no significant difference between ICG and non-ICG groups may be seen effectively. For example, the current results show no patients who switch to open surgery in the ICG group. In contrast, two patients in the non-ICG group have changed from laparoscopic surgery to open surgery. Still, the difference in the proportion of switching to open surgery between the two groups is not statistically significant. Third, the average follow-up time of this study is relatively short. Although the current results suggest that the proportion of residual lymph nodes in the non-ICG group was higher, no significant difference was found in the local recurrence of tumors between the two groups during the 1-year follow-up. Overall, reliably defining the efficacy and safety of ICG fluorescence-guided LPND in patients with rectal cancer nephropathy requires a longitudinal study in a large sample size.

In conclusion, this study shows that ICG fluorescence-guided imaging is a feasible and convenient technology to assist LPND because it has unique advantages in improving short-term outcomes. ICG fluorescence guidance technology is simple to operate, which is worth popularizing.

## Data availability statement

The raw data supporting the conclusions of this article will be made available by the authors, without undue reservation.

## Ethics statement

The studies involving human participants were reviewed and approved by the Ethics Committee of Fengrun District People's Hospital of Tangshan. Written informed consent for participation was not required for this study in accordance with the national legislation and the institutional requirements.

## Author contributions

J-YD: had full access to all the data in the study and take responsibility for the integrity of the data and the accuracy of the data analysis, concept, and design. Z-JH, J-DW, and B-SL: acquisition, analysis, or interpretation of data. J-YL and Y-CW: drafting of the manuscript. All authors contributed to the article and approved the submitted version.

## Funding

This work was supported by the Medical Science Research Project of Hebei Province in 2021 (No: 20211342). The sponsor or funding organization had no role in the design or conduct of this research.

## Conflict of interest

The authors declare that the research was conducted in the absence of any commercial or financial relationships that could be construed as a potential conflict of interest.

## Publisher's note

All claims expressed in this article are solely those of the authors and do not necessarily represent those of their affiliated organizations, or those of the publisher, the editors and the reviewers. Any product that may be evaluated in this article, or claim that may be made by its manufacturer, is not guaranteed or endorsed by the publisher.
